# A volume-controlled, anatomy-driven autoplanning strategy for whole-pelvic volumetric modulated arc therapy

**DOI:** 10.3389/fonc.2026.1836562

**Published:** 2026-05-07

**Authors:** Chih-Yuan Lin, An-Cheng Shiau, Ti-Hao Wang, Shih-Ming Hsu

**Affiliations:** 1 Department of Biomedical Imaging and Radiological Sciences, National Yang Ming Chiao Tung University, Taipei, Taiwan; 2 Medical Physics and Radiation Measurements Laboratory, National Yang Ming Chiao Tung University, Taipei, Taiwan; 3 Department of Biomedical Imaging and Radiological Science, China Medical University, Taichung, Taiwan; 4 Department of Radiation Oncology, China Medical University Hospital, Taichung, Taiwan; 5 Department of Medicine, China Medical University, Taichung, Taiwan

**Keywords:** automated treatment planning, RapidPlan, volume-controlled AutoPlan, volumetric modulated arc therapy, whole pelvic radiotherapy

## Abstract

**Background and purpose:**

Whole pelvic radiotherapy (WPRT) involves volumetric modulated arc therapy (VMAT) planning that requires careful consideration of target coverage and organ-at-risk (OAR) sparing. Manual planning is labor-intensive and subject to inter-planner variability. To address these challenges, we developed and evaluated a Volume-Controlled Autoplan (VCAP), an anatomy-driven automated planning strategy that incorporates volumetric overlap information into the optimization framework.

**Materials and methods:**

This study retrospectively analyzed a cohort of patients with pelvic malignancies who underwent WPRT. VCAP was compared against clinical planning (CP), Autoplan (AP), and RapidPlan (RP). Dosimetric endpoints included PTV coverage (V_95_, conformity index, homogeneity index) and OAR sparing (rectum, bladder, bowel bag, and femoral heads). Regression models based on OAR–PTV overlap were used to derive predefined thresholds for guiding the optimization process. Dose-volume histograms (DVHs) and dose distributions were evaluated, and statistical comparisons were performed.

**Results:**

VCAP achieved comparable PTV coverage relative to CP, AP, and RP, with V_95_ consistently above 95%. Rectal doses were reduced, with V_30_ decreased by an average of 24.6% relative to CP and 12-13% relative to AP and RP. Bladder sparing was also improved in the 15–40 Gy range. The bowel bag showed lower intermediate-dose exposure, with V_30_ reduced by more than 20.5% compared with CP. Planning efficiency was markedly enhanced, with mean planning time reduced from approximately 120 minutes with CP to 21 minutes with automatic strategies, while VCAP demonstrated consistent plan quality across the cohort.

**Conclusion:**

VCAP provides an efficient and anatomy-driven approach to VMAT planning for WPRT. By incorporating volumetric overlap constraints into the optimization process, VCAP enhances OAR sparing while preserving robust PTV coverage. These findings suggest that VCAP may improve planning efficiency and consistency in clinical practice. Further validation is warranted to assess its generalizability and potential role in broader clinical applications.

## Introduction

Whole pelvic radiotherapy (WPRT) is a cornerstone in managing common pelvic malignancies but is associated with significant toxicities to surrounding organs at risk (OARs) (1–3). Manual Volumetric Modulated Arc Therapy (VMAT) planning is time-consuming and subject to inter-planner variability, which compromises plan quality and poses challenges for standardizing treatments in multicenter trials. While automated planning has emerged to improve efficiency and consistency, several studies show that challenges remain (4, 5). Previous studies have investigated automated planning approaches for WPRT (6–8).

While automated planning has improved consistency, existing approaches may have limited flexibility in adapting to patient-specific anatomical variations, particularly in cases with substantial overlap between OARs and the planning target volume (PTV). In this context, an interpretable, anatomy-driven strategy may be useful for guiding optimization according to patient-specific geometry.

The Simultaneous Integrated Protection (SIP) technique manages significant target-OAR overlap by defining a subvolume within the PTV that receives a reduced dose, enabling dose modulation to spare critical structures without compromising overall target coverage. Its successful application in various cancers makes it a key component in complex treatment planning (9). This approach has been applied in cervical cancer (6), SBRT for pancreatic ductal adenocarcinoma (10), and liver cancer (11). Geometric relationships between targets and OARs, including overlap-related features, have been widely used in knowledge-based planning approaches such as RapidPlan (RP), where spatial information contributes to dose prediction. In this context, we developed a SIP-inspired planning strategy, termed Volume-Controlled Autoplan (VCAP), to explore a structured and interpretable approach to automated planning. This strategy incorporates a simple and interpretable linear regression model using the OAR–PTV overlap ratio, a geometry-based parameter associated with dose distribution, to guide anatomy-aware optimization. By quantifying this relationship, VCAP supports a patient-specific planning approach and provides a more interpretable framework for dose optimization.

As a complementary strategy to improve dose conformity, a Half-Beam (HB) technique was also incorporated into the automated plan design (12). Accordingly, this study aimed to develop and evaluate three automated VMAT treatment planning strategies for WPRT: a script-based autoplan (AP), a knowledge-based RapidPlan (RP), and a SIP-inspired VCAP. These automated plans were systematically compared with clinically delivered plans (CP) to evaluate dosimetric performance and planning efficiency.

## Materials and methods

### Patient selection

This retrospective study included 584 patients with pelvic malignancies who underwent WPRT to 45 Gy in 25 fractions at the Department of Radiation Oncology, China Medical University Hospital, between August 2010 and December 2024. Patients with prior pelvic irradiation were excluded. Following CT simulation, target volumes and OARs were contoured according to Radiation Therapy Oncology Group (RTOG) guidelines. Because this was a retrospective study spanning 2010-2024, all contours were derived from clinically approved treatment plans and retrospectively reviewed for consistency according to institutional contouring guidelines before analysis. This study was approved by the Institutional Review Board of China Medical University Hospital (CMUH113-REC1-144). All procedures were conducted in accordance with relevant guidelines and regulations. The requirement for informed consent was waived by the IRB owing to the retrospective nature of the study and the use of de-identified data.

### Data source and model development

**Figure 1:**
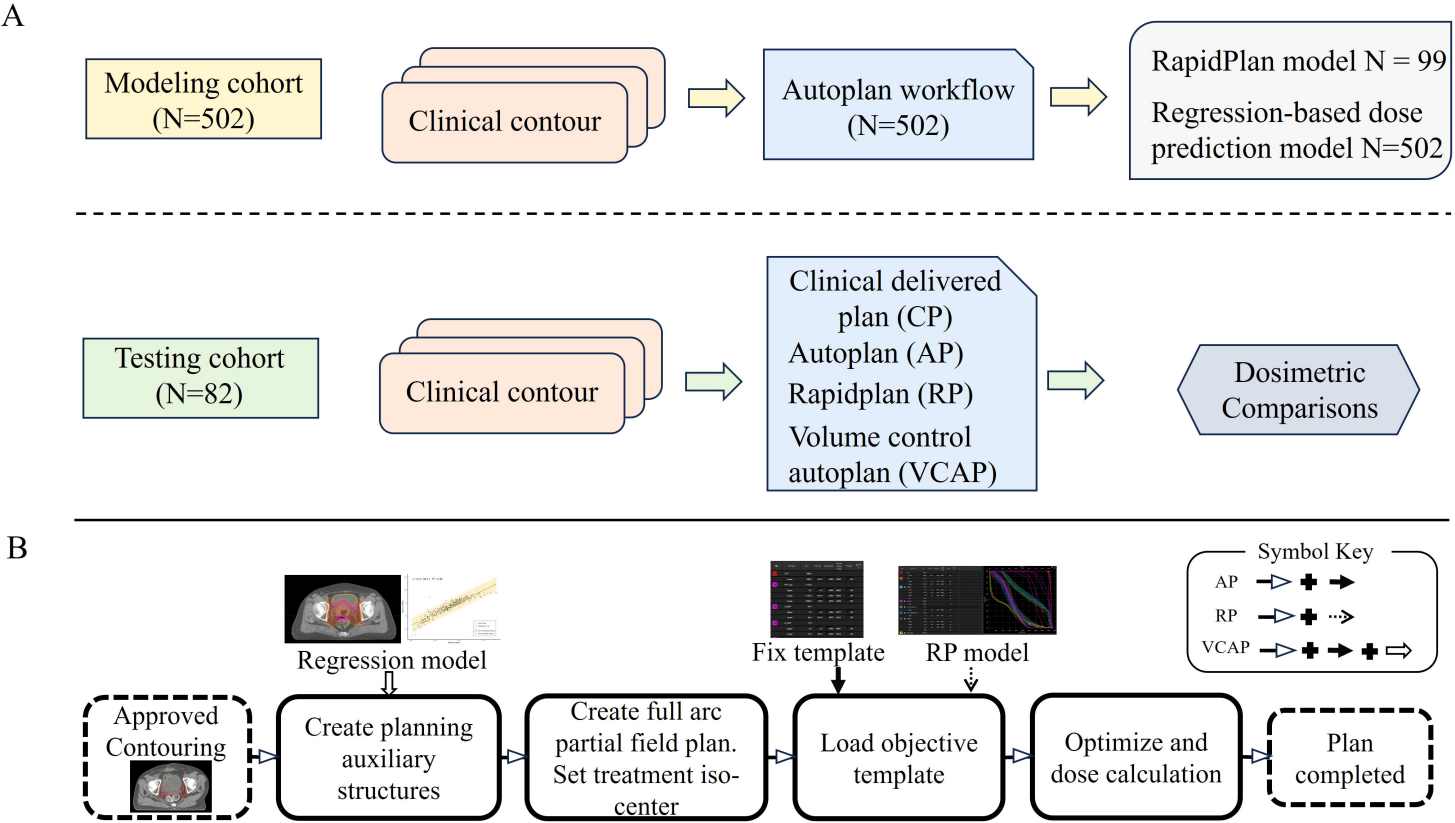
Workflow for model development and testing of automated VMAT planning strategies. **(A)** modeling was performed using 502 contours to establish the AP workflow, which generated both a RP model (N = 99) and a regression model (N = 502). Testing employed 82 contours to evaluate four strategies: CP, AP, RP, and VCAP. **(B)** treatment planning steps included auxiliary structure generation, beam setup, optimization, dose calculation, and final plan generation. Specific structures were derived using the regression model. Optimization was guided either by predefined templates or DVH predictions from the RP model. Arrows indicate the information flow, with different symbols representing CP, AP, RP, and VCAP strategies (as shown in the figure legend). CP, clinical plan; AP, AutoPlan; RP, Rapid Plan; VCAP, volume control AutoPlan.

A total of 584 clinical cases were retrospectively collected and divided into a modeling cohort (n = 502) and a testing cohort (n = 82). An AP system using PyESAPI generated two models from the modeling cohort: a knowledge-based RP model (n=99) and a regression-based dose prediction model (n=502). The testing cohort (n=82) was used to evaluate four planning strategies: the CP, AP, RP, and VCAP. The overall workflow is illustrated in **Figure 1A**.

### Automated VMAT planning strategies (AP, RP, and VCAP)

All automated VMAT plans were generated using the Eclipse TPS, version 18 (Varian Medical Systems Inc., Palo Alto, California, USA), for a 10 MV TrueBeam linear accelerator. Plan optimization was performed using the Photon Optimizer (PO) with jaw tracking enabled, convergence mode turned on, and graphics processing unit (GPU) acceleration. The Anisotropic Analytical Algorithm (AAA) served as the dose calculation engine.

**Figure 2:**
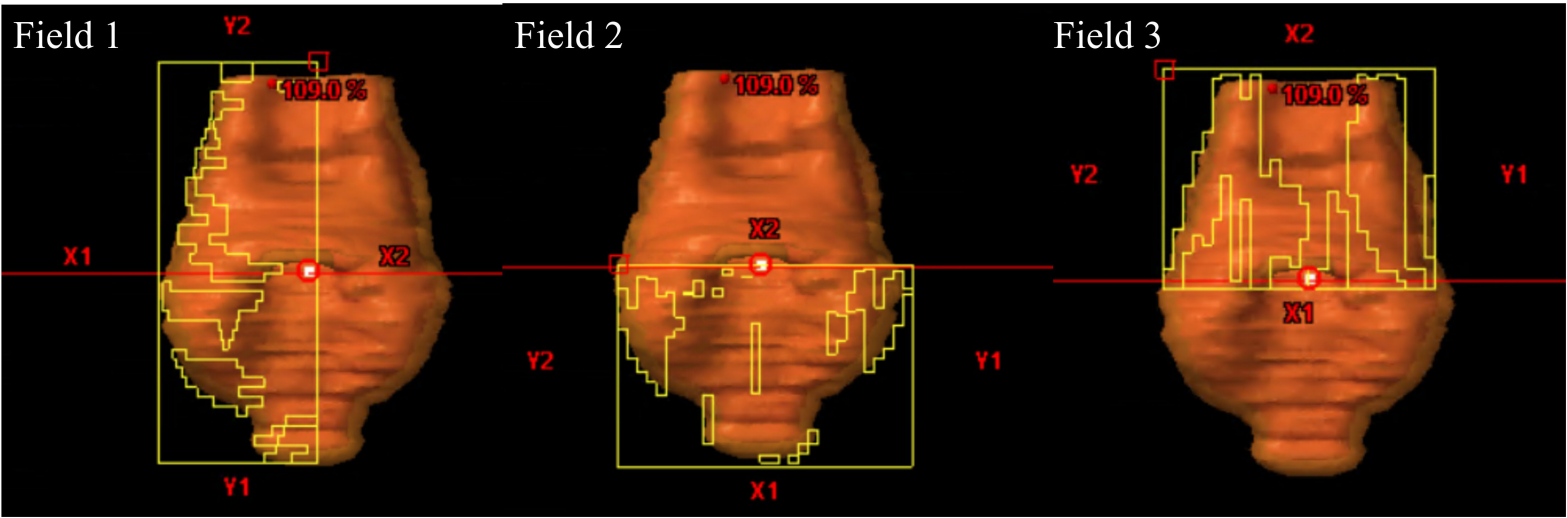
A three-arc VMAT plan was generated using the half-beam technique. The first arc was delivered from 179° to 181° (358° rotations) with a collimator angle of 0°, with the field fitted to the PTV and either X1 or X2 closed to 0 cm. The second arc was delivered from 181° to 179° with a collimator angle of 90°, with the field fitted to the PTV and X2 opened to 1 cm. The third arc was delivered from 179° to 181°, also with a collimator angle of 90°, with the field fitted to the PTV and X1 opened to 1 cm.

The standard AP workflow utilized a script to automate the creation of auxiliary planning structures (**Supplementary Table 1**), create a three-arc VMAT plan emulating a half-beam technique and set the isocenter (**Figure 2**), apply a predefined objective template (**Supplementary Table 2**), and run the optimization and calculation. The workflow is illustrated in **Figure 1B**. Using this workflow, 502 AP plans were generated.

Two models were developed from the 502 AP VMAT plans in the modeling cohort: 1) a knowledge-based RP model trained on a curated subset of 99 high-quality plans, and 2) linear regression models for each OAR trained on the full cohort. The 99 plans used for RP training were selected from the 502 AP-generated plans based on overall dosimetric quality, including acceptable target coverage, OAR sparing, and compliance with institutional planning objectives. A curated subset was used for RP training to provide a homogeneous, high-quality training dataset, which is important for knowledge-based model performance. Previous studies have suggested that while a minimum of approximately 20 cases is sufficient to establish a RP model, improved performance is generally observed when 60–120 cases are used for training. In this context, the sample size used in the present study falls within the recommended range for robust model development (13, 14).

The VCAP workflow used regression-derived thresholds to support an anatomy-driven planning approach. The OAR-PTV overlap ratio was defined as the overlapping volume between the OAR and the PTV divided by the total OAR volume: V(OAR ∩ PTV)/V(OAR). If this ratio exceeded predefined thresholds, auxiliary target-related structures were generated for optimization. Specifically, an optimization target volume (PTVopt) was defined to prioritize target coverage outside OAR-overlap regions and overlap structures between the contracted PTV (cPTV) and each OAR (OAR-OL-cPTV) were generated to explicitly account for target-OAR overlap regions. These structures were combined to form the VCAP planning target volume (PTV_VCAP_), which served as the central reference structure for generating additional auxiliary structures, including concentric ring structures and cropped OAR structures, for dose shaping during optimization. In this study, “OL” denotes the overlap region between an OAR and the contracted PTV, whereas structures prefixed with “C-” denote cropped OAR structures generated by excluding the PTV_VCAP_ plus a 5-mm margin. The definitions and planning purposes of these auxiliary structures are summarized in **Supplementary Table 3**, and the corresponding optimization objectives are provided in **Supplementary Table 4**.

**Figure 3:**
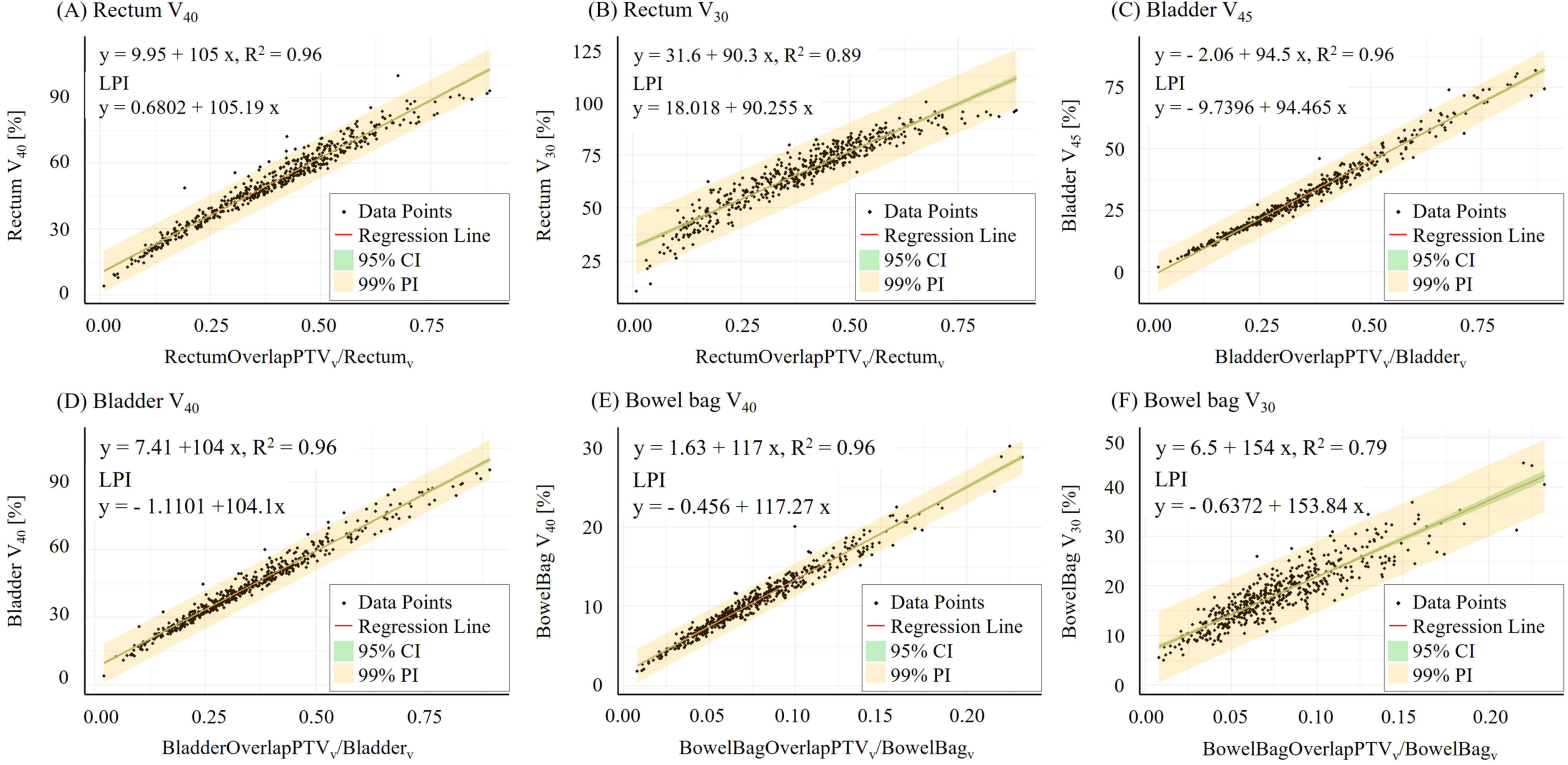
Linear regression model between OAR-PTV overlap ratio and dose-volume parameters across 502 APs. **(A)** rectum V_40_, **(B)** rectum V_30_, **(C)** bladder V_45_, **(D)** bladder V_40_, **(E)** bowel bag V_40_, and **(F)** bowel bag V_30_. Scatter plots show the relationship between OAR-PTV overlap ratio and V_x_ for rectum, bladder, and bowel bag. Regression lines (red), 95% confidence intervals (green), and 99% prediction intervals (yellow) are shown. Strong correlations were observed, with R² ≥ 0.89 for most metrics. LPI, lower prediction interval formula; CI, confidence interval; PI, prediction interval.

These thresholds were derived from the lower prediction interval (LPI) of the regression models (**Figure 3**). Specifically, for each OAR, the institutional dose-volume constraint was substituted into the LPI equation, and the corresponding overlap ratio was solved as the threshold for triggering PTV_VCAP_-based structure generation. The detailed LPI equations and threshold derivation are provided in **Supplementary Table 5**. For example, the LPI equation for rectum V_40Gy_ was y = 105.19 x + 0.68. Setting y=35, corresponding to the institutional rectum V_40Gy_ constraint of 35%, yielded an overlap ratio threshold (x) of 0.33. The final thresholds were 0.33 for the rectum, 0.39 for the bladder, and 0.26 for the bowel bag.

The design differences among the three automated VMAT planning strategies are summarized in **Table 1**. All plans used a standardized three-arc VMAT with half-beam design, but they differed in predictive information, auxiliary-structure design, and optimization objectives.

**Table 1:** Key design features of automated VMAT planning strategies.

Design feature	AP	RP	VCAP
Beam arrangement	3-arc VMAT with half-beam technique	Same as AP	Same as AP
Auxiliary-structure design	Multiple derived auxiliary structures	Simplified structure set; no overlap-based cropping required	Anatomy-driven auxiliary-structure adaptation using SIP-inspired principles
Planning objectives	Predefined objectives	DVH prediction using RP model	Predefined objectives with anatomy-driven, SIP-inspired adaptation

AP, AutoPlan; RP, Rapid Plan; VCAP, Volume Control AutoPlan.

### Plan evaluation

All plans (CP, AP, RP, VCAP) were normalized such that 95% of the PTV received 100% of the prescription dose. Dosimetric comparisons focused on the PTV and OARs using predefined dose constraints (15–17) listed in **Table 2**. Plans were evaluated for PTV coverage using the conformity index (CI) and homogeneity index (HI), and for dose fall-off using the gradient index (GI_x_), defined at threshold levels of 40 Gy, 30 Gy, and 20 Gy to evaluate dose gradient beyond the PTV margins (6). OAR doses, monitor units (MU) per field, and planning time were also compared. Planning time was defined as the total time required to generate a treatment plan, measured from plan initialization to completion of the final dose calculation, including auxiliary structure generation, beam setup, objective/constraint preparation, optimization, and plan normalization, but excluding contouring time. The same definition of planning time was applied to all planning strategies (CP, AP, RP, and VCAP). Therefore, the reported planning time should be interpreted as a workflow-level efficiency metric rather than an isolated optimization/computation time.

**Table 2:** Dosimetric comparison of PTV and OARs among CP, AP, RP, and VCAP plans. Values are presented as mean ± standard deviation (median).

Structure	Parameters	Constraints	CP	AP	RP	VCAP
PTV	V_95%_ (%)	≥ 95%	99.71 ± 0.21 (99.80)	99.86 ± 0.06 (99.90)	99.89 ± 0.05 (99.90)	99.50 ± 0.36 (99.6)
	V_0.03cc_ (%)	< 110%	108.76 ± 1.30 (108.85)	108.13 ± 0.76 (108.00)	108.62 ± 0.71(108.60)	109.24 ± 1.12 (109.00)
	CI		0.89 ± 0.02 (0.90)	0.87 ± 0.01 (0.87)	0.94 ± 0.00 (0.94)	0.86 ± 0.02 (0.86)
	HI		0.08 ± 0.01 (0.08)	0.07 ± 0.01 (0.07)	0.07 ± 0.00 (0.07)	0.08 ± 0.01 (0.08)
bladder	V_45Gy_ (%)	< 35%	28.07 ± 11.34 (27.05)	28.87 ± 11.02 (27.35)	27.58 ± 9.96 (26.75)	26.45 ± 6.82 (26.40)
	V_40Gy_ (%)	< 40%	45.60 ± 12.59 (45.25)	41.55 ± 12.19 (39.95)	40.31 ± 11.90 (39.25)	38.51 ± 8.35 (38.90)
	V_0.03cc_ (%)	< 105%	106.76 ± 1.18 (106.70)	104.49 ± 0.61 (104.4)	106.39 ± 0.75 (106.30)	105.20 ± 0.86 (105.10)
rectum	V_40Gy_ (%)	< 35%	54.16 ± 12.29 (54.70)	49.99 ± 10.97 (50.50)	47.95 ± 10.81 (48.45)	39.29 ± 5.31 (39.30)
	V_30Gy_ (%)	< 60%	75.59 ± 11.48 (75.80)	65.72 ± 10.76 (67.05)	65.04 ± 10.33 (66.75)	57.03 ± 7.10 (56.70)
	V_0.03cc_ (%)	< 105%	106.80 ± 1.31 (106.80)	104.68 ± 0.86 (104.60)	106.44 ± 0.86 (106.40)	105.19 ± 1.06 (105.10)
bowel bag	V_45Gy_ (cc)	< 195 cc	111.91 ± 38.40 (105.90)	86.34 ± 29.26 (85.35)	101.14 ± 33.99 (100.80)	87.76 ± 29.35 (86.60)
	V_40Gy_ (%)	< 30%	9.43 ± 3.96 (8.95)	7.53 ± 3.01 (7.25)	7.95 ± 3.20 (7.55)	7.40 ± 2.96 (7.10)
	V_30Gy_ (%)	< 40%	18.62 ± 7.20 (17.20)	14.76 ± 5.21 (14.10)	15.19 ± 5.37 (14.50)	14.80 ± 5.21 (14.30)
	V_0.03cc_ (%)	< 110%	107.39 ± 1.21 (107.30)	105.00 ± 0.77 (105.00)	107.33 ± 0.64 (107.25)	105.78 ± 1.04 (105.65)
femoral head_R	V_45Gy_ (%)	< 50%	0.19 ± 1.46 (0.00)	0.06 ± 0.30 (0.00)	0.03 ± 0.23 (0.00)	0.09 ± 0.31 (0.00)
	V_30Gy_ (%)	< 15%	10.84 ± 6.24 (10.40)	12.98 ± 4.90 (12.40)	1.62 ± 1.90 (1.05)	12.60 ± 4.95 (12.25)
femoral head_L	V_45Gy_ (%)	< 50%	0.09 ± 0.24 (0.00)	0.03 ± 0.08 (0.00)	0.00 ± 0.00 (0.00)	0.08 ± 0.21 (0.00)
	V_30Gy_ (%)	< 15%	12.63 ± 5.86 (12.60)	12.63 ± 3.99 (12.30)	1.65 ± 1.51 (1.30)	11.59 ± 4.31 (11.40)
GI	GI_40Gy_		1.38 ± 0.04 (1.37)	1.35 ± 0.03 (1.36)	1.28 ± 0.03 (1.29)	1.34 ± 0.03 (1.34)
	GI_30Gy_		2.24 ± 0.13 (2.25)	2.11 ± 0.07 (2.11)	1.93 ± 0.09 (1.95)	2.07 ± 0.07 (2.08)
	GI_20Gy_		4.40 ± 0.35 (4.40)	4.10 ± 0.23 (4.13)	3.91 ± 0.28 (3.92)	4.01 ± 0.23 (4.02)
MU/field			243.94 ± 40.38 (243.62)	280.64 ± 13.02 (281.90)	306.15 ± 14.26 (308.45)	300.13 ± 11.80 (301.22)

CP, Clinical Plan; AP, AutoPlan; RP, Rapid Plan; VCAP, Volume Control AutoPlan.

### Statistical analysis

The relationship between OAR-PTV overlap and dose was evaluated using linear regression. In addition to fitted equations and coefficients of determination (R²), the fitted regression coefficients, standard errors, adjusted R² values, and model *p*-values were summarized to further characterize model robustness. LPI equations were additionally used to derive overlap-ratio thresholds for VCAP triggering. Dosimetric parameters across the four planning groups were compared using the Friedman test, with *post hoc* Wilcoxon signed-rank tests applying Holm *p*-value adjustments. Statistical significance was set at *p* < 0.05. Analyses were performed using R Statistics Version 4.4.3.

## Results

### Cohort composition

A total of 584 patients were included in this study, comprising a modeling cohort of 502 patients and a testing cohort of 82 patients. The dataset represented a heterogeneous mix of pelvic malignancies, including 323 patients with cervical cancer, 165 with endometrial cancer, and 96 with prostate cancer. Regarding disease stage, 33 patients had unknown stage, whereas 190, 193, 125, and 43 patients were classified as stage I, II, III, and IV, respectively.

### Linear regression model

Contours from 502 clinical treatment plans were processed using AP. A strong linear correlation was observed between the volumetric overlap ratio of OARs with the PTV and their corresponding dose-volume parameters (V_x_), as shown in **Figure 3**. The overlap ratio was consistently associated with dose exposure across all examined OARs: rectum (R² > 0.89), bladder (R² > 0.96), and bowel bag (R² > 0.79). Additional model summary statistics, including fitted coefficients, standard errors, adjusted R² values, model *p*-values, and LPI equations for threshold derivation, are provided in **Supplementary Table 5**.

For the rectum and bladder, the relationship between overlap ratio and V_40_ or V_45_ was particularly robust, with R² values reaching up to 0.96. These findings indicate a nearly linear increase in OAR dose with increasing OAR-PTV overlap fraction. The bowel bag, while showing slightly greater interpatient variability, especially at lower dose levels, still maintained strong correlation at moderate and higher doses, with R² up to 0.96 for V_40_ and 0.79 for V_30_.

Across all models, the 95% confidence and prediction intervals supported the robustness and reliability of the fits. Collectively, these findings support the use of the volumetric overlap ratio as an interpretable geometry-based parameter associated with OAR dose exposure in pelvic radiotherapy planning.

### Dosimetric comparisons

**Figure 4:**
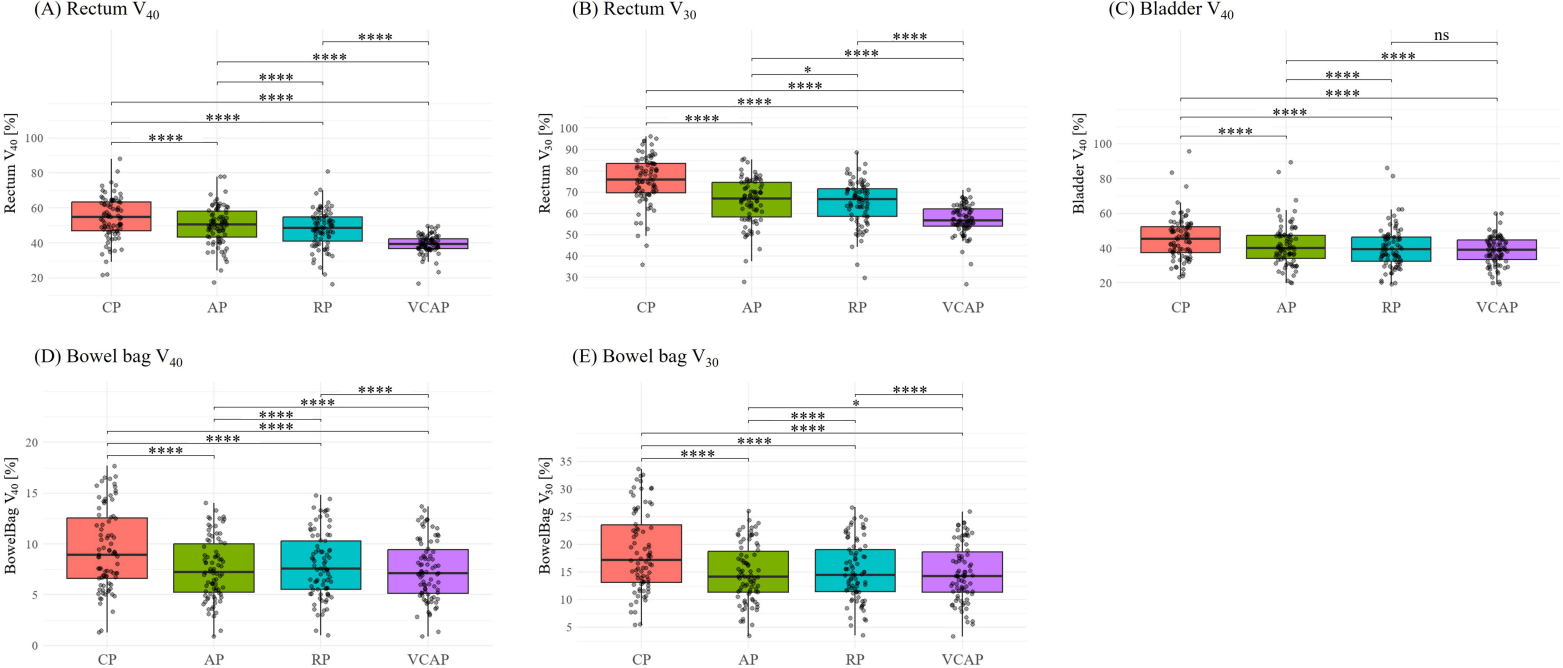
Dose comparison of OAR across four planning techniques. Boxplots show the percentage volume of **(A)** rectum V_40_, **(B)** rectum V_30_, **(C)** bladder V_40_, **(D)** bowel bag V_40_, and **(E)** bowel bag V_30_ for CP, AP, RP, and VCAP plans. Statistical significance is denoted by asterisks as follows: ns, not significant; (*) *p* < 0.05; (****) *p* < 0.0001. CP, clinical plan; AP, AutoPlan; RP, Rapid Plan; VCAP, volume control AutoPlan.

The dosimetric comparison results are summarized in **Table 2** and illustrated in **Figure 4**. All automated VMAT treatment planning strategies (AP, RP, and VCAP) demonstrated improved or more consistent dosimetric performance relative to the manually optimized CP in target coverage, conformity, and OAR sparing. Among the automated approaches, RP showed the highest target coverage (V_95%_: 99.89 ± 0.05%) and the highest conformity index (CI: 0.94 ± 0.01, *p* < 0.0001), indicating precise dose sculpting. Although HI remained consistent across plans (~0.08), dose heterogeneity was significantly reduced in RP and AP (*p* < 0.0001).

VCAP showed favorable reductions in bladder and rectal dose (e.g., bladder V_45Gy_: 26.45% ± 6.82 vs. CP: 28.07% ± 11.34, *p* < 0.0001). Bowel sparing was comparable among strategies, though CP showed slightly higher values. Femoral head doses remained within tolerance across all plans.

GI_x_ analysis showed a steeper dose fall-off in RP (e.g., GI_40Gy_: 1.28 ± 0.03), whereas CP showed the more gradual gradients (GI_40Gy_: 1.38 ± 0.04). The increased MU observed in the autoplanned plans likely reflects the higher modulation complexity of the automated planning strategies, which may have contributed to the improved conformity and OAR sparing.

Several OAR-related dosimetric parameters showed significant differences across the four planning strategies. In particular, VCAP achieved the lowest values for rectum V_40Gy_ and V_30Gy_, bladder V_40Gy_, and bowel bag V_40Gy_ and V_30Gy_. In contrast, RP showed favorable conformity and gradient performance. To better isolate the contribution of the volume control strategy, an additional focused comparison between AP and VCAP was performed under otherwise identical planning conditions. Under these conditions, the addition of volume control resulted in improved OAR sparing while maintaining comparable target coverage. In particular, VCAP achieved lower rectum dose-volume parameters, with rectum V_40Gy_ reduced from 49.99% to 39.29% and V_30Gy_ from 65.72% to 57.03%. Similar improvements were observed for the bladder, with reductions in V_45Gy_ and V_40Gy_. Bowel dose parameters showed comparable or slightly improved values. These improvements are consistent with the use of auxiliary-structure design and optimization objectives described in **Supplementary Tables 3** and **4**.

In addition to summary statistics, paired comparisons were performed using patient-level differences (Δ = AP - CP). Improvements were not uniform across all cases. For example, AP achieved lower rectum V_30Gy_ than CP in 85.4% of patients, while higher values were observed in 14.6%. For bladder V_40Gy_, improvements were observed in 80.5% of patients, whereas 19.5% showed higher values. In contrast, for femoral head dose parameters, AP did not consistently demonstrate improvement relative to CP, with a subset of cases showing higher values. These findings indicate that the observed dosimetric differences reflect improved consistency rather than uniform superiority.

### Stage-based subgroup analysis of VCAP

To further assess the robustness of the proposed VCAP approach across clinically relevant subgroups, a stage-based dosimetric subgroup analysis was performed for VCAP plans in the testing cohort (**Supplementary Table 6**). The 82 cases were stratified into three categories, namely unknown stage, stage I-II, and stage III-IV, to represent different levels of tumor burden. Overall, target coverage and OAR sparing remained broadly comparable across stage groups, without evidence of substantial stage-specific deterioration in plan quality. In particular, PTV coverage was highly consistent, with V_95%_ ranging from 99.46% to 99.62%. Similarly, bladder V_40Gy_ and rectum V_30Gy_ showed only modest variation across stage groups: bladder V_40Gy_ ranged from 36.92% to 39.49% (difference, 2.57 percentage points), and rectum V_30Gy_ ranged from 54.94% to 58.80% (difference, 3.86 percentage points). Although bowel bag V_30Gy_ showed relatively greater variation, ranging from 12.09% to 16.17% (difference, 4.08 percentage points), the overall dosimetric pattern remained stable across stage categories. These findings suggest that VCAP maintained stable dosimetric performance across the stage subgroups.

### Plan consistency, reproducibility and efficiency

As shown in **Table 2**, all automated VMAT treatment planning strategies showed improved plan consistency compared with CP, with lower standard deviations across key metrics. RP had the most stable dose gradients (GI_40Gy_: 1.28 ± 0.03; GI_30Gy_: 1.93 ± 0.09; GI_20Gy_: 3.91 ± 0.28), while VCAP showed minimal variability in rectum V_30Gy_ (57.03 ± 7.10%). In contrast, CP showed higher variability, including rectum V_30Gy_ SD = 11.48% and bladder V_40Gy_ SD = 12.59%.

These patterns are consistent with the dosimetric comparisons shown in **Table 2**, in which the SIP-inspired VCAP approach achieved substantial reductions in several OAR dose metrics relative to CP, while femoral head doses remained within acceptable clinical limits.

The automated planning approach required only 21 minutes to complete, compared to 120 minutes for the CP, representing an 82.5% reduction in planning time. Based on the workflow-level definition described in the Methods, this substantial time saving improved workflow efficiency across cases.

### DVH and dose distribution

**Figure 5:**
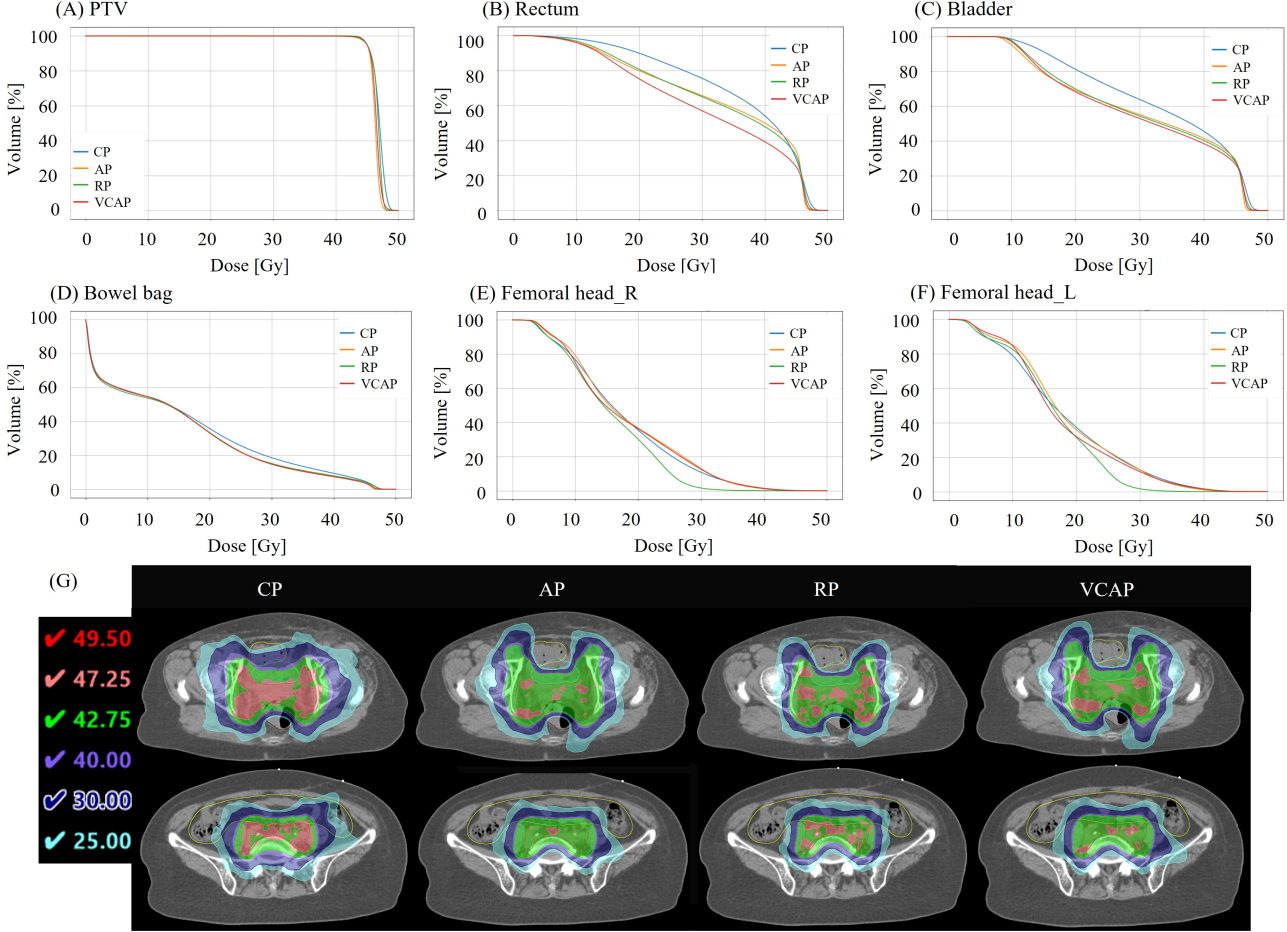
Average DVHs **(A-F)** and representative dose distributions **(G)** comparing CP with automated VMAT treatment planning strategies (AP, RP, and VCAP). Automatic planning maintained PTV coverage while significantly improving OAR sparing, and resulted in fewer high-dose regions within the PTV. Rectal and bladder doses were notably reduced in the 15–45 Gy range, with VCAP achieving the lowest rectal dose. For the bowel bag, all automatic strategies resulted in lower doses than CP, particularly in the 15–40 Gy range. Femoral head doses were comparable across techniques and remained within clinical constraints. CP, clinical plan; AP, AutoPlan; RP, Rapid Plan; VCAP, volume control AutoPlan.

Average DVH (**Figures 5A–F**) and dose distribution (**Figure 5G**) analysis revealed notable improvements in OAR sparing with automated VMAT treatment planning strategies (AP, RP, VCAP), while maintaining comparable PTV coverage across all plans. All techniques achieved steep dose fall-off near the prescription level with minimal inter-plan variation (**Figure 5A**).

Automated VMAT treatment planning strategies reduced rectal doses, especially within the 15-45 Gy range, with VCAP demonstrating lower dose levels (**Figure 5B**). Similarly, bladder sparing was improved, with VCAP showing notable dose reduction between 15-40 Gy (**Figure 5C**). For the bowel bag, all automatic planning techniques consistently resulted in lower dose exposure compared to CP, particularly in the 15-40 Gy range (**Figure 5D**).

Femoral head doses were comparable across all techniques and remained within clinical dose constraints. Overall, automated VMAT treatment planning strategies were associated with improved OAR sparing in the low- to mid-dose regions while preserving target coverage.

## Discussion

This study showed that automated VMAT planning strategies improved plan consistency and OAR sparing compared with manual planning for WPRT. Notably, the SIP-inspired VCAP approach showed favorable dosimetric performance, supporting the value of integrating patient-specific anatomical features into the planning process. The observed performance of the automated plans may also have been influenced, at least in part, by the use of a three-arc HB technique, which is known to minimize low-dose spillage in pelvic radiotherapy (12, 16).

The paired analysis further showed that improvements were not uniform across all cases, with a subset of patients demonstrating higher dose values for certain OAR parameters. This finding supports that the observed differences reflect improved consistency rather than universal superiority. The observed improvements in automated planning may be attributed to the consistent application of predefined optimization templates and auxiliary-structure design, which reduce inter-planner variability and enable more standardized dose optimization. Importantly, these findings should not be interpreted as indicating that clinical plans are of inferior quality. Rather, clinical plans may reflect variability in planning practice and practical constraints in routine workflows, including time limitations and differences in planning priorities. In addition, differences in beam arrangement and planning configuration may also contribute to the observed dosimetric variations. In this study, automated plans were generated using a standardized three-arc half-beam design, whereas clinical plans may have involved different arc configurations depending on planner preference and clinical considerations.

A key feature of VCAP is its use of a simple, interpretable linear regression model based on the OAR-PTV overlap ratio. While complex dose prediction models exist (18–20), our approach provides a practical and interpretable geometry-based parameter for guiding dose optimization, enabling preemptive plan adaptation without extensive computational resources. Previous studies have rarely implemented SIP principles within such a quantitative, anatomy-driven approach. By translating the SIP concept into a clinically applicable algorithm, this regression-guided method enables patient-specific, spatially adaptive dose shaping without manual tuning.

The use of auxiliary structures, including the optimization target volume, OAR-PTV overlap regions, and concentric ring structures, enables explicit control of spatial dose distribution during optimization. By separating non-overlapping and overlapping target regions and introducing geometrically defined constraints, the VCAP framework provides a structured and interpretable approach to automated planning. Compared with conventional planning approaches, the proposed framework emphasizes geometric interpretability and consistency in dose shaping, rather than introducing fundamentally new predictive features. In this context, the proposed VCAP approach differs from existing methods by explicitly integrating geometry-based overlap information into the optimization process through dynamic auxiliary-structure generation. In particular, when the OAR–PTV overlap ratio exceeds predefined thresholds, additional auxiliary structures are adaptively generated to guide optimization. Optimization objectives are then applied to these cropped or overlap-aware structures rather than uniformly to the entire OAR, enabling more explicit spatial control of dose distribution in anatomically challenging regions.

An important finding of this study is the trade-off between OAR sparing and conformity-related performance among the automated planning strategies. VCAP achieved the greatest reductions in bladder and rectal dose, whereas RP demonstrated superior target conformity and steeper dose fall-off. These results suggest that the relative advantage of each strategy may depend on the clinical priority. VCAP may be advantageous when sparing of adjacent OARs is emphasized, whereas RP may be advantageous when conformity or gradient metrics are prioritized. Rather than indicating universal superiority of one method, these findings highlight distinct optimization characteristics of the two approaches.

The success of the RP model, trained on consistent AP-generated plans rather than variable clinical plans, highlights the potential of model distillation. This approach can create efficient and robust knowledge-based models even without a large library of historical, manually-created plans (21). Although RP was not as aggressive as the VCAP in terms of OAR sparing, it maintained good target coverage and dose homogeneity. Furthermore, it significantly reduced planning time, which is a key advantage in high-throughput clinical settings. In addition, studies that applied multi-criteria optimization (MCO) have demonstrated the benefit of region-specific dose modulation by distinguishing between overlapping and non-overlapping areas of the PTV and OARs. These findings highlight the broader potential of incorporating spatially aware planning strategies into model-guided treatment planning (22).

Although the present study focused on dosimetric rather than clinical outcome endpoints, the observed reductions in rectal, bladder, and bowel dose may still be clinically meaningful, because dose-volume exposure of these organs has been associated with GI and GU toxicity in pelvic radiotherapy. In particular, rectal and bowel dose-volume parameters have been linked to GI toxicity, while bladder dose-volume effects have been associated with GU toxicity. However, the magnitude of clinical benefit depends on the specific organ, dose-volume parameter, and extent of dose reduction, and dosimetric improvement alone does not constitute direct evidence of reduced toxicity (3, 23, 24).

Given the heterogeneous composition of the dataset, the generalizability of the findings across disease sites should be interpreted with caution. Nevertheless, stage-based subgroup analysis demonstrated broadly consistent dosimetric performance of VCAP across the limited/advanced stage subgroups, supporting its robustness within the studied cohort.

This study has several limitations, including its single-institution design. More importantly, the comparison in this study was intended to evaluate different planning strategies rather than to isolate the effect of individual technical parameters. Importantly, the AP-versus-VCAP comparison provides a more focused assessment of the contribution of volume control. The two approaches were implemented under otherwise identical planning conditions, with the intended difference being the use of volume control. The observed reductions in rectum and bladder dose therefore suggest that the volume control strategy itself contributed to improved OAR sparing. In this context, beam arrangement was considered part of the overall planning strategy, together with the optimization framework, constraint design, and workflow implementation. Therefore, the observed dosimetric differences in the overall workflow comparisons should be interpreted as reflecting the performance of the complete planning approaches rather than the isolated effect of any single planning component. Similarly, the reported reduction in planning time should be interpreted as a workflow-level efficiency gain rather than an isolated optimization/computation-time advantage, because different planning strategies involved different degrees of manual interaction and automation.

These findings suggest that the clinical value of automated planning may lie not only in potential dosimetric improvement, but also in improving planning consistency, reducing inter-planner variability, and enhancing workflow efficiency in routine practice. Despite their demonstrated advantages, automated planning strategies are not yet uniformly adopted in routine practice, as their implementation requires site-specific model development, workflow integration, validation, and clinical acceptance. However, ongoing developments in automation and standardization are expected to facilitate broader clinical adoption in the future. Future studies with standardized technical settings may help further clarify the specific contribution of each component within the planning strategy.

## Conclusion

The SIP-inspired VCAP strategy, implemented with an HB technique, showed favorable potential for OAR sparing in WPRT while maintaining robust target coverage. With its reproducibility and adaptability, this strategy may be useful for improving plan consistency and planning efficiency in clinical practice.

## Data Availability

The original contributions presented in the study are included in the article/**Supplementary Material**. Further inquiries can be directed to the corresponding authors.
