# Supplementary material for: A volume-controlled, anatomy-driven autoplanning strategy for whole-pelvic volumetric modulated arc therapy
Source: Front Oncol. 2026 May 7;16:1836562. doi: 10.3389/fonc.2026.1836562 (PMC13189775; doi:10.3389/fonc.2026.1836562)
Supplement: Supplementary file 1 [file Table1.docx]

Supplementary Material

Supplementary Tables

**Table 1.** Definitions and generation methods of auxiliary structures used in the AP workflow.

| Auxiliary structures | Method |
| --- | --- |
| Optimization target volume (PTV_opt_) | Generated by subtracting all three OARs (bladder, rectum, and bowel bag) from the PTV. |
| Overlap regions between OARs and PTV (OARs-OL-PTV) | Overlap between PTV and each OAR. |
| Outer ring around PTV (OuterRing) | Ring structure from 1 cm to 5 cm outside the PTV. |
| Inner ring around PTV (InnerRing) | Ring structure from 1 mm to 1 cm outside the PTV. |
| Target dose-control structure (ConTD) | PTV expanded by 5 mm minus CTV. |
| Cropped OAR structures (C-OARs) | OARs cropped excluding PTV+5 mm margin |
| 20% isodose-based control structure (Con20^*^) | Two fields (AP and PA) were added, covering the full PTV in the Y-direction and 4 cm wide in the X-direction, each delivering 100 MU. The 20% isodose region was generated and subtracted from the PTV expanded by 1 cm. |

^*^ Inspired by Yu et al., who introduced avoidance structures (AS) in HB VMAT to improve OAR sparing by controlling dose spillage [1], our study incorporated a similar strategy in the automated VMAT Planning.

Abbreviations: OAR, organ at risk; PTV, planning target volume; CTV, clinical target volume; OL, overlap region; ConTD, target dose-control structure. Structures prefixed with “C-” denote cropped OAR structures generated by excluding the PTV plus a 5-mm margin.

**Table 2.** Dose optimization objectives used in the AP planning template.

| Structure | Type | Volume [%] | Dose [Gy] | Priority |
| --- | --- | --- | --- | --- |
| CTV | Lower | 100 | 45.9 | 200 |
| PTV_opt_ | Upper | 0 | 46.35 | 300 |
|  | Lower | 100 | 45 | 250 |
|  | Lower | 99 | 45.45 | 230 |
| Body | Upper | 0 | 46.35 | 250 |
| OuterRing | Upper | 0 | 36.00 | 140 |
| ConTD | Upper | 0 | 45.45 | 300 |
| InnerRing | Upper | 0 | 45.0 | 200 |
| Con20 | Upper | 0 | 22.50 | 150 |
|  | Upper | 10 | 13.50 | 100 |
|  | Upper | 30 | 5.0 | 100 |
| C-bladder | Upper | 0 | 22.50 | 110 |
|  | Upper | 10 | 13.50 | 100 |
|  | Upper | 30 | 5.0 | 100 |
| C-rectum | Upper | 0 | 22.50 | 110 |
|  | Upper | 10 | 13.50 | 100 |
|  | Upper | 30 | 5.0 | 100 |
| C-bowelbag | Upper | 0 | 22.50 | 120 |
|  | Upper | 10 | 13.50 | 100 |
|  | Upper | 30 | 5.0 | 100 |
| Bladder-OL-PTV | Lower | 100 | 45.00 | 200 |
|  | Upper | 0 | 45.45 | 250 |
| Rectum-OL-PTV | Lower | 100 | 45.00 | 200 |
|  | Upper | 0 | 45.45 | 250 |
| BowelBag-OL-PTV | Lower | 100 | 45.00 | 200 |
|  | Upper | 0 | 45.45 | 250 |
| Femoral_Head_L | Upper | 5 | 30.00 | 100 |
| Femoral_Head_R | Upper | 5 | 30.00 | 100 |

Abbreviations: CTV, clinical target volume; PTV_opt_, Optimization target volume; OAR, organ at risk; OL, overlap region. “C-” indicates cropped structures excluding PTV + 5 mm margin.

**Table 3.** Definitions and generation methods of auxiliary structures used in the VCAP planning workflow.

| Auxiliary structures | Definition / Generation Method | Planning Purpose |
| --- | --- | --- |
| Optimization target volume (PTV_opt_) | Generated by subtracting all three OARs (bladder, rectum, bowel bag) from the PTV. | Prioritize target coverage in non-overlapping regions |
| Overlap regions between OARs and contracted PTV (OARs-OL-cPTV) | Overlap between cPTV and each OAR. | Control dose in target-OAR overlap regions |
| VCAP planning target volume (PTV_VCAP_) | The union of PTV_opt_ and OARs-OL-cPTV | Reference structure for auxiliary structure generation. |
| Outer ring around PTV_VCAP_ (OuterRing_VCAP_) | Ring structure from 1 cm to 5 cm outside the PTV_VCAP_ | Control intermediate dose spillage |
| Inner ring around PTV_VCAP_ (InnerRing_VCAP_) | Ring structure from 1 mm to 1 cm outside the PTV_VCAP_ | Improve conformity |
| Target dose-control structure (ConTD_VCAP_) | PTV_VCAP_ expanded by 5 mm minus CTV. | Control high-dose distribution |
| Cropped OAR structures (C-OARs_VCAP_) | OARs cropped excluding PTV_VCAP_+5 mm margin | Applies constraints to non-overlapping OAR regions. |
| 20% isodose-based control structure (Con20_VCAP_*) | Two fields (AP and PA) were added, covering the full PTV in the Y-direction and 4 cm wide in the X-direction, each delivering 100 MU. The 20% isodose region was generated and subtracted from the PTV_VCAP_ expanded by 1 cm | Control low-dose spread |

^*^ Inspired by Yu et al., who introduced avoidance structures (AS) in HB VMAT to improve OAR sparing by controlling dose spillage [1], our study incorporated a similar strategy in the automated VMAT Planning.

Abbreviations: OAR, organ at risk; PTV, planning target volume; cPTV, contracted planning target volume; VCAP, Volume-Controlled Autoplan; OL, overlap region; PTVopt, optimization target volume; ConTD, target dose-control structure. “C-” denotes cropped structures generated by excluding the PTV_VCAP_ plus a 5-mm margin.

**Table 4.** Dose optimization objectives used in the VCAP planning template.

| Structure | Type | Volume [%] | Dose [Gy] | Priority |
| --- | --- | --- | --- | --- |
| CTV | Lower | 100 | 45.9 | 200 |
| PTV_opt_ | Upper | 0 | 46.35 | 300 |
|  | Lower | 100 | 45 | 250 |
|  | Lower | 99 | 45.45 | 230 |
| Body | Upper | 0 | 46.35 | 250 |
| OuterRing_VCAP_ | Upper | 0 | 36.00 | 140 |
| ConTD_VCAP_ | Upper | 0 | 45.45 | 300 |
| InnerRing_VCAP_ | Upper | 0 | 45.0 | 200 |
| Con20_VCAP_ | Upper | 0 | 22.50 | 150 |
|  | Upper | 10 | 13.50 | 100 |
|  | Upper | 30 | 5.0 | 100 |
| C-bladder_VCAP_ | Upper | 0 | 22.50 | 110 |
|  | Upper | 10 | 13.50 | 100 |
|  | Upper | 30 | 5.0 | 100 |
| C-rectum_VCAP_ | Upper | 0 | 22.50 | 110 |
|  | Upper | 10 | 13.50 | 100 |
|  | Upper | 30 | 5.0 | 100 |
| C-bowelbag_VCAP_ | Upper | 0 | 22.50 | 120 |
|  | Upper | 10 | 13.50 | 100 |
|  | Upper | 30 | 5.0 | 100 |
| Bladder-OL-cPTV | Lower | 100 | 45.00 | 200 |
|  | Upper | 0 | 45.45 | 250 |
| Rectum-OL-cPTV | Lower | 100 | 45.00 | 200 |
|  | Upper | 0 | 45.45 | 250 |
| BowelBag-OL-cPTV | Lower | 100 | 45.00 | 200 |
|  | Upper | 0 | 45.45 | 250 |
| Femoral_Head_L | Upper | 5 | 30.00 | 100 |
| Femoral_Head_R | Upper | 5 | 30.00 | 100 |

**Abbreviations:** CTV, clinical target volume; PTV_opt_, Optimization target volume; OAR, organ at risk; OL, overlap region; cPTV, contracted planning target volume; VCAP, Volume-Controlled Autoplan. “C-” indicates cropped structures excluding PTV_VCAP_ + 5 mm margin.

**Table 5**. Linear regression model parameters and LPI-based threshold derivation for OAR dose prediction

| Endpoint | Parameters | SE (Intercept) | SE (Slope) | R² | Adjusted R² | Model *p*-value | LPI equation | Derived overlap-ratio threshold |
| --- | --- | --- | --- | --- | --- | --- | --- | --- |
| Rectum V_40Gy_ | <35% | 0.41 | 0.97 | 0.958 | 0.958 | <0.0001 | y = 105.19x + 0.68 | 0.33 |
| Rectum V_30Gy_ | <60% | 0.60 | 1.42 | 0.887 | 0.887 | <0.0001 | y = 90.26x + 18.02 | 0.47 |
| Bladder V_40Gy_ | <40% | 0.36 | 0.94 | 0.960 | 0.960 | <0.0001 | y = 104.10x − 1.11 | 0.39 |
| Bladder V_45Gy_ | <35% | 0.32 | 0.84 | 0.960 | 0.960 | <0.0001 | y = 94.47x − 9.74 | 0.47 |
| Bowel bag V_40Gy_ | <30% | 0.09 | 1.01 | 0.963 | 0.963 | <0.0001 | y = 117.27x − 0.46 | 0.26 |
| Bowel bag V_30Gy_ | <40% | 0.29 | 3.45 | 0.794 | 0.794 | <0.0001 | y = 153.84x − 0.64 | 0.26 |

**Abbreviations:** SE, standard error; OAR, organ at risk; LPI, lower prediction interval.

**Table 6**. Stage-based subgroup analysis of dosimetric parameters for VCAP plans in the testing cohort. Values are presented as mean ± standard deviation.

| Structure | Parameters | Constraints | Overall (N=82) | Unknown(N=10) | I-II (N=44) | III-IV (N=28) |
| --- | --- | --- | --- | --- | --- | --- |
| PTV | V_95%_ (%) | ≥ 95% | 99.50 ± 0.36 | 99.62 ± 0.27 | 99.46 ± 0.36 | 99.53 ± 0.37 |
|  | V_0.03cc_ (%) | < 110% | 109.24 ± 1.12 | 108.89 ± 0.88 | 109.37 ± 1.32 | 109.17 ± 0.83 |
|  | CI |  | 0.86 ± 0.02 | 0.88 ± 0.02 | 0.86 ± 0.02 | 0.86 ± 0.01 |
|  | HI |  | 0.08 ± 0.01 | 0.08 ± 0.01 | 0.08 ± 0.01 | 0.08 ± 0.01 |
| bladder | V_45Gy_ (%) | < 35% | 26.45 ± 6.82 | 26.58 ± 9.24 | 27.06 ± 6.36 | 25.43 ± 6.71 |
|  | V_40Gy_ (%) | < 40% | 38.51 ± 8.35 | 38.66 ± 10.56 | 39.49 ± 7.88 | 36.92 ± 8.29 |
|  | V_0.03cc_ (%) | < 105% | 105.20 ± 0.86 | 104.75 ± 0.41 | 105.33 ± 1.03 | 105.14 ± 0.60 |
| rectum | V_40Gy_ (%) | < 35% | 39.29 ± 5.31 | 38.22 ± 2.66 | 40.59 ± 4.29 | 37.62 ± 6.83 |
|  | V_30Gy_ (%) | < 60% | 57.03 ± 7.10 | 55.07 ± 4.15 | 58.80 ± 6.04 | 54.94 ± 8.72 |
|  | V_0.03cc_ (%) | < 105% | 105.19 ± 1.06 | 104.81 ± 0.60 | 105.38 ± 1.29 | 105.01 ± 0.66 |
| bowel bag | V_45Gy_ (cc) | < 195 cc | 87.76 ± 29.35 | 80.00 ± 26.12 | 94.11 ± 29.88 | 80.57 ± 28.16 |
|  | V_40Gy_ (%) | < 30% | 7.40 ± 2.96 | 5.88 ± 2.67 | 8.23 ± 3.03 | 6.63 ± 2.58 |
|  | V_30Gy_ (%) | < 40% | 14.80 ± 5.21 | 12.09 ± 5.13 | 16.17 ± 5.19 | 13.63 ± 4.75 |
|  | V_0.03cc_ (%) | < 110% | 105.78 ± 1.04 | 105.39 ± 0.77 | 105.94 ± 1.15 | 105.67 ± 0.89 |
| femoral head_R | V_45Gy_ (%) | < 50% | 0.09 ± 0.31 | 0.30 ± 0.85 | 0.04 ± 0.08 | 0.10 ± 0.15 |
|  | V_30Gy_ (%) | < 15% | 12.60 ± 4.95 | 14.53 ± 9.33 | 12.82 ± 4.16 | 11.56 ± 3.80 |
| femoral head_L | V_45Gy_ (%) | < 50% | 0.08 ± 0.21 | 0.08 ± 0.19 | 0.11 ± 0.26 | 0.04 ± 0.11 |
|  | V_30Gy_ (%) | < 15% | 11.59 ± 4.31 | 12.24 ± 3.81 | 12.25 ± 4.56 | 10.30 ± 3.89 |
| GI | GI_40Gy_ |  | 1.34 ± 0.03 | 1.33 ± 0.02 | 1.33 ± 0.03 | 1.34 ± 0.03 |
|  | GI_30Gy_ |  | 2.07 ± 0.07 | 2.06 ± 0.06 | 2.06 ± 0.07 | 2.09 ± 0.08 |
|  | GI_20Gy_ |  | 4.01 ± 0.23 | 4.02 ± 0.27 | 3.96 ± 0.21 | 4.09 ± 0.24 |
| MU/field |  |  | 300.13 ± 11.80 | 297.06 ± 6.28 | 298.31 ± 12.69 | 304.08 ± 11.07 |

Reference

1. Yu PC, Wu CJ, Nien HH, Lui LT, Shaw S, Tsai YL. Half-beam volumetric-modulated arc therapy in adjuvant radiotherapy for gynecological cancers. J Appl Clin Med Phys. 2022;23(1):e13472.
